# “Shoulder pain and limitation of motion in a young girl: think different”

**DOI:** 10.1186/s13052-022-01332-4

**Published:** 2022-07-30

**Authors:** Matteo Trevisan, Luca Di Lenarda, Serena Pastore, Alessia Saccari, Gianluca Canton, Umberto Lucangelo, Andrea Taddio, Luigi Murena

**Affiliations:** 1 Department of Medicine, Surgery, and Health Sciences, University of Trieste, Via dell’Istria 65/1, 34100 Trieste, Italy; 2 Institute for Maternal and Child Health, IRCCS “Burlo Garofolo”, Trieste, Italy; 3 Orthopaedics and Traumatology Unit, Cattinara Hospital, Azienda Sanitaria Universitaria Giuliano-Isontina (ASUGI), University of Trieste, Trieste, Italy; 4 Department of Perioperative Medicine, Intensive Care and Emergency, Cattinara Hospital, Trieste University, Trieste, Italy

**Keywords:** Synovial chondromatosis, Shoulder pain, Cartilage metaplasia, Loose bodies, Rice-grain pattern, Arthroscopy

## Abstract

**Background:**

Primary Synovial Chondromatosis (PSC) is a rare benign tumor of the synovial membrane in which cartilage metaplasia produces calcific loose bodies within the articular space. Only a few cases are reported in the pediatric population and its etiology remains unknown. This condition typically affects large weight-bearing joints with pain, swelling and decrease range of motion. Due to its slow progressions, delayed diagnosis is frequent and differential diagnosis should consider other chronic arthritis and malignancies. While arthroscopic removal of loose bodies is the current treatment up to now, the association of partial or complete synovectomy is debated.

**Case presentation:**

We report about a 14-year-old girl with a long-lasting right shoulder pain, especially during movements or exercise, localized tenderness and hypotonia of the glenohumeral joint. No previous trauma was mentioned. Blood exams, Mantoux test and plain radiography of the right shoulder were unremarkable. Ultrasound imaging revealed echogenic and calcified bodies stretching the glenohumeral joint and dislocating the long head of biceps tendon. Magnetic resonance showed a “rice-grain” pattern of the right shoulder. From an arthroscopic surgery, multiple loose white bodies were removed within the synovial membrane, and synovial chondromatosis was confirmed by histological analysis. At one month follow up visit, the patient completely recovered without pain.

**Conclusion:**

Synovial chondromatosis is a very uncommon cause of mono articular pain in children, especially when it affects shoulder. Pediatricians should keep in mind this condition to avoid delayed diagnosis and treatment, even in consideration of the low risk of malignant transformation. Through this case, we would highlight common diagnostic pitfalls and treatment of synovial chondromatosis.

**Supplementary Information:**

The online version contains supplementary material available at 10.1186/s13052-022-01332-4.

## Background

Primary synovial chondromatosis (PSC) is a rare cause of arthralgia in children [1–4]. SC is characterized by intra-articular loose bodies due to synovial metaplasia, but its pathogenesis remains unknown [5, 6]. PSC typically affects large and weight-bearing joints, especially knees, hips and rarely the shoulders. Patients may be asymptomatic, and symptoms can develop slowly with localized pain, limited range of motion, swelling or locking of the affected joint as main manifestations. Laboratory exams and plain radiography may be unremarkable or misleading, while magnetic resonance imaging is considered the gold standard for diagnosis [7, 8]. A prompt recognition could avoid secondary damage to the joint. To date, surgical arthroscopy remains the best treatment available, but relapses and malignant transformation, although rare, are described. Here, we report a case of synovial chondromatosis of the right shoulder, highlighting common pitfalls in its differential diagnosis and treatment.

## Case presentation

**Fig. 1:**
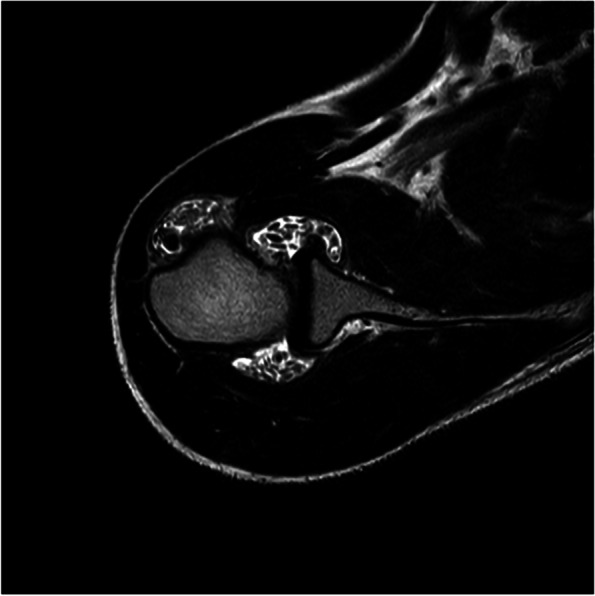
T2-weighted MRI shows a pronounced effusion of the shoulder joint, especially the subacromial bursa and axillary recess. Multiple hypointense lesions are present within the articular space

A previously healthy 14-year-old girl was referred to our rheumatologic clinic for a seven-month history of pain of the right shoulder. No previous trauma was mentioned, and family history was unremarkable for chronic arthritis or other autoimmune diseases. For this reason, she stopped sports activities. The patient complained about localized pain in her right shoulder, especially during movements and no morning stiffness was documented. Treatment with non-steroidal anti-inflammatory drugs (NSAIDs) was administered, which was ineffective. Physical examination showed tenderness to palpation of the long head of the right biceps and subacromial region with hypotonia of the glenohumeral joint. Blood exams and Mantoux test resulted negative as well as a plain radiograph. An ophthalmological evaluation, a chest X-Ray and abdominal ultrasound were unremarkable. Given the persistence of shoulder pain and motion limitation, the patient underwent ultrasound imaging that revealed a laterally dislocated long head of biceps tendon (LHBT) surrounded by echogenic and minute calcified bodies, stretching the synovial sheath with an axial diameter of 2.5 cm. A subsequent Magnetic Resonance Imaging (MRI) showed a stretched shoulder joint, especially in the subacromial bursa and axillary recess, and multiple minute hypointense lesions were appreciated within the articular cavity (Fig. 1).

**Fig. 2:**
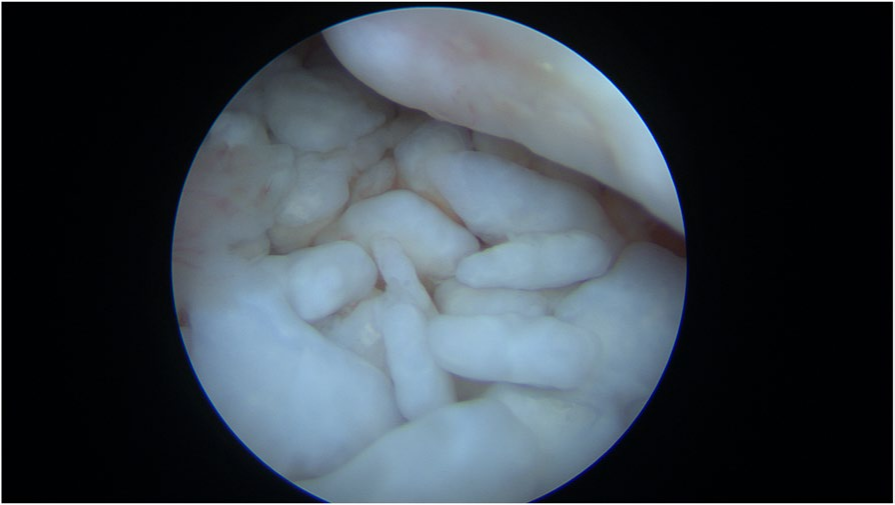
Arthroscopy of the right shoulder: white calcific loose bodies within the articular space

**Fig. 3:**
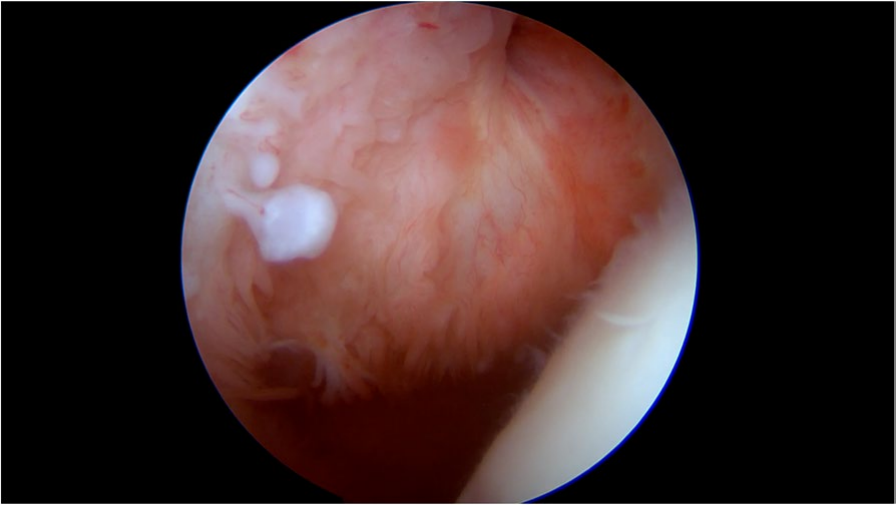
A sessile cartilaginous body arising from the synovial membrane

**Fig. 4:**
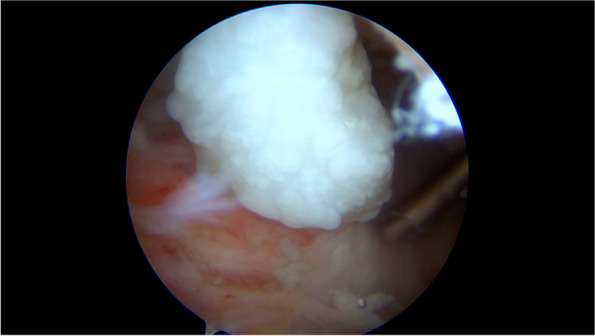
A cartilaginous loose body from the opening of the LHBT sheath

A synovial chondromatosis was suspected, therefore the patient underwent arthroscopic surgery of the right shoulder under general anesthesia and peripheral nerve block. In the intra-articular phase of arthroscopic surgery, multiple white, shiny loose bodies were found within the articular space and were removed along with the inflamed synovial membrane (Fig. 2, an additional movie file shows this in more detail [see Additional file 1]). A few sessile cartilaginous bodies of the synovial membrane were also noted and sent for histological examination (Fig. 3, additional file 1). The LHBT appeared healthy, and no loose bodies could be retrieved in the intraarticular, most cranial portion of the bicipital groove. Based on the MRI imaging findings, arthroscopic exploration of the subacromial space was implemented. Opening of the LHBT sheath revealed the presence of abundant loose bodies, presenting with the same macroscopic aspects of the intra-articular bodies whereas of bigger dimensions. LHBT sheath synovitis was also detected (Fig. 4, an additional movie file shows this in more detail [see Additional file 2]). Histological examination of the loose bodies revealed multiple islets of hyaline cartilage and foci of calcification with chronic inflammation of the synovia, confirming the diagnosis of PSC.

The patient was discharged the day after surgical treatment without anesthesiologic or other surgical complications. Post-operative indications included right shoulder rest in an arm sling for 10 days, with active-assisted ROM (Range of Motion) allowed as tolerated. At one month follow-up, the patient recovered full range of motion without complaining of pain in the shoulder and she did not show recurrence at 6 months.

## Discussion and conclusion

Synovial chondromatosis is a rare benign condition affecting the synovial membrane, with only a few cases described in pediatric population [1–4]. It is characterized by intra-articular loose bodies deriving from the subsynovial tissue, in which metaplastic synovial cells form cartilaginous bodies which can even ossify. Traditionally, chondromatosis can be classified as primary, if a previously healthy joint is affected, or secondary, if preceded by a pathological condition. The former is idiopathic and typical of the younger age [5, 6]. The natural history of the disease encompasses three stages: in stage I, an early inflamed synovia is present; stage II is characterized by cartilaginous sessile formation, while in stage III there are calcified loose bodies with minimal synovial inflammation [9].

Symptoms may be aspecific and insidious, as in our case, with a slow worsening of pain, tenderness, decreased range of motion and locking of the affected joint. Nevertheless, in a few cases, it can be asymptomatic and found incidentally. SC usually affects large and weight-bearing joints, mainly the knee or hip, while ankle, shoulder and elbow are extremely rare.

Plain radiographs can address the right diagnosis and multiple intra-articular calcified lesions are pathognomonic, with the typical “rice-grain” pattern, but in 20% of cases standard radiography can be unremarkable, as in our patient*.* Indeed, Maurice et al. showed that only 54% of nodules in the acute phase of disease were visible on plain radiographs, whereas 88% of transitional phase and 100% of mature phase nodules could be seen on radiographic imaging[7]. For all these reasons, delayed diagnosis and treatment are common. MRI or computed tomography (CT) can identify the typical lesions, even in the early stages. Early MRI findings show multiple hypointense formations in T1-weight sequences, which are hyperintense in T2 for the high water-content of cartilaginous bodies within the articular space, bursae or along the tendon sheaths. A contrast-enhanced synovia characterizes the first stages of the disease. If loose bodies are yet to be ossified, hypointense lesions are present in all the sequences [8].

In children, persistent arthralgia requires an extended work-up. Pediatric long-lasting mono- or oligo-arthralgia should comprise juvenile idiopathic arthritis (JIA) in the differential diagnosis [10]. Oligoarticular JIA typically affects large joints with swelling, pain and limitations of movements. Laboratory exams and plain radiographs may be negative, but the elevation of inflammatory markers and anti-nuclear antibodies (ANA) are typical. Considering 20% of JIA are associated to uveitis, an ophthalmological evaluation can help in the final diagnosis [11]. In our patient, as commonly observed in mechanical pain, symptoms were mainly present during upper arm movements, while inflammatory pain typical of arthritis arises after resting with morning stiffness. Moreover, shoulder involvement is rare in JIA as well as it usually manifests during childhood. Finally, ultrasound may be helpful in JIA diagnosis since it may detect synovial hypertrophy and doppler-power positivity.

Of note, mono-articular involvement with similar insidious pain requires to rule out tubercular arthritis. The slow progression of symptoms is similar, and both can show the “rice-grain” radiological pattern [12, 13]. Lastly, SC belongs to a wide group of neoplastic-like lesions of the articular space. MRI can help distinguish chondromatosis from other benign lesions such as pigmented villo-nodular synovitis or synovial hemangioma [2, 6]. Nevertheless, a differential diagnosis with intra-articular malignancies may be more insidious. For example, chondrosarcoma shares similar radiological findings and histological confirmation is mandatory. The clinical indications to arthroscopy in children are rare, but the presence of an uncommon, unexplained mono-articular arthritis is one of the recommendations.

Pediatricians should be aware of this condition because a prompt diagnosis and treatment can avoid secondary damage to the joint, especially in the puberal and developmental ages. Moreover, retrospective studies from adults described a low risk of developing chondrosarcoma (5% of SC) [14–16].

Although in sporadic case reports, conservative management was described, the main treatment remains surgery, removing arthroscopically the loose bodies [2, 4, 17]. Up to now, there is no strong evidence about the role of synovectomy, even if this technique is preferred due to a lower risk of relapses [18], that unfortunately remain frequent. After surgery, the patient should be followed up with MRI for at least two years to rule out recurrence.

In conclusion, this case shows a rare condition of monoarticular pain in children, and pediatricians should keep in mind synovial chondromatosis in the differential diagnosis to avoid delayed diagnosis and treatment. This can be helpful to prevent articular damage and to rule out malignancies.

## Supplementary Information

**Additional file 1.** Shoulder arthroscopy, intra-articular view from posterior portal. Multiple white, shiny loose bodies can be seen within the articular space, especially in the axillary recess. Loose bodies are removed with a shaver. Inflamed synovial tissue is retrieved in all areas, removal with grasper and ablation with radiofrequency is demonstrated. A tissue grasper is also used to remove the sessile cartilaginous bodies forming on the synovial membrane.

**Additional file 2.** Shoulder arthroscopy, subacromial view from antero-lateral portal. The LHBT sheath is identified and opened, with retrieval of abundant loose bodies, some of which present with bigger dimensions than the intra-articular side. LHBT sheath synovitis can also be detected.

## Data Availability

Not applicable.

## Abbreviations

ANA: Anti-Nuclear Antibodies
CT: Computed Tomography
JIA: Juvenile Idiopathic Arthritis
LHBT: Long Head of Biceps Tendon
MRI: Magnetic Resonance Imaging
NSAIDs: Non-Steroidal Anti-Inflammatory Drugs
PSC: Primary Synovial Chondromatosis
ROM: Range of Motion
